# Supplementary material for: Quantile Regression for Longitudinal Functional Data with Application to Feed Intake of Lactating Sows
Source: J Agric Biol Environ Stat. 2024 Feb 6;30(1):211–30. doi: 10.1007/s13253-024-00601-5 (PMC11885350; doi:10.1007/s13253-024-00601-5)
Supplement: Supplementary file 1 — (html 2448 KB) [file 13253_2024_601_MOESM1_ESM.html]

fQGAM: DTI data example


Code 

- Show All Code
- Hide All Code

# fQGAM: DTI data example

### Supplementary material to *Quantile regression for longitudinal functional data with application to feed intake of lactating sows*

#### M.L. Battagliola, H. Sørensen, A. Tolver, A.-M. Staicu

#### April 2023

- Data preparation
- Fitting fQGAM and computing predicted quantiles
- Bootstrap
  - Block bootstrap
  - Wild bootstrap
- Bias-adjustment and confidence intervals
- Fitting a time-homogeneous model

In this document we show how to implement fQGAM using the DTI dataset available in \(\texttt{R}\) package \(\texttt{refund}\).

Given data \(\{(Y\_{ij},X\_{ij}(\cdot), t\_{ij})\}\_{ij}\) where \(i=1,\dots, N\) (subjects) and \(j=1,\dots, n\_i\) (observations for each subject), the model we are interested in is \[Q\_{Y\_{ij}| {X}\_{ij}, u\_i}^\tau(t\_{ij}) = \alpha^\tau(t\_{ij}) + \int\_{\mathcal{S}} \beta^\tau(s,t\_{ij}) {X}\_{ij}(s)ds + u\_i.\] Here, \(\tau\in (0,1)\) is a fixed quantile level, and we assume curves \(\{X\_{ij}(\cdot)\}\) are \(L^2(\mathcal{S})\), where we take \(\mathcal{S}=[0,1]\). We refer to the manuscript for details.

## Data preparation

```
# Preliminaries
library(refund)
library(qgam)
library(ggplot2)
library(gridExtra)

theme_set(theme_bw())
```

The DTI dataset is loaded below. We are going to use \(\texttt{cca}\) as covariates, \(\{X\_{ij}(\cdot)\}\) and \(\texttt{pasat}\) as response, \(\{Y\_{ij}\}\), and \(\texttt{visit.time}\) as longitudinal time stamps \(\texttt{t\_ij}\). For convenience, we first select those observations that do not have missing data in \(\texttt{cca}\) only include those related to patients that were checked more than once:

```
data(DTI)
myData <- DTI[complete.cases(DTI$cca),]
myData <- subset(myData, Nscans>1)
dim(myData$cca)
```

```
## [1] 334  93
```

```
N <- dim(myData$cca)[1]
S <- dim(myData$cca)[2]
```

We then prepare the matrices used for the integration with respect to \(s\) (the functional coordinate) as well as \(t\) (the longitudinal time):

```
sVec <- seq(0,1,length=S) 
sGrid <- matrix(sVec,N,S, byrow=TRUE)
```

```
tVec <- myData$visit.time
tGrid <- matrix(tVec,N,S, byrow=FALSE)
maxT <- max(tVec)
```

We now prepare the data.frame used for the call to \(\texttt{qgam}\):

```
myDataFit <-data.frame(y=myData$pasat,
                       id=factor(myData$ID),
                       time=myData$visit.time)
myDataFit$Xobs <- myData$cca
myDataFit$sMat <- sGrid
myDataFit$tMat <- tGrid

Nid <- length(unique(myDataFit$id))
```

Finally, we extract the 20% and 80% pointwise quantiles of \(\texttt{cca}\), which we will later use for prediction. For illustration, we plot the \(\texttt{cca}\) curves along with the two quantiles curves (the 20% in blue and the 80% in red). Notice that we do not perform any prior smoothing of \(\{X\_{ij}(\cdot)\}\) as there are no missing values and the grid is fine (S=93).

```
cca20 <- apply(myData$cca,2,quantile, probs=0.2)
cca80 <- apply(myData$cca,2,quantile, probs=0.8)
```

```
X <- nb <- f <- NULL
for(i in 1:N){
  X <- c(X, myData$cca[i,])
  nb <- c(nb, rep(i,S))
  f <- c(f, rep(1,S))
}

data.plot <- data.frame(X=c(X,cca20,cca80),
                        nb=factor(c(nb, rep(i+1,S), rep(i+2,S))),
                        s=rep(sVec,(N+2)),
                        curve=factor(c(f,rep("cca20",S), rep("cca80",S)))
)

ggplot(data.plot, aes(x=s,y=X,group=nb, color=curve)) + geom_line()   + scale_color_manual(values=c("f"="gray", "cca20"="blue", "cca80"="red"), limits=c("cca20", "cca80")) + labs(color = "") + ylab(expression(paste(X[ij](s)))) + theme(legend.position="bottom")
```

## Fitting fQGAM and computing predicted quantiles

We choose quantile level \(\tau=0.1\) and fit the model. The model formula is defined in terms of standard GAM syntax, and we can thus choose the type and size of bases flexibly. Notice that the by option is used to get the integral term in the model.

```
tau <- 0.1

formula <- as.formula('y ~ s(time, bs="cr", k=10) + 
                      te(sMat, tMat, by=Xobs, bs=c("cr", "cr"), k=c(10,10)) + 
                      s(id, bs="re")')

fit <- qgam(formula, qu=tau, data=myDataFit)
```

```
## Estimating learning rate. Each dot corresponds to a loss evaluation. 
## qu = 0.1........................done
```

```
# Number of coefficients not related to random effects
Nfixed <- length(fit$coefficients) - Nid

# Variance-covariance matrix for fixed effects parameters
cov <- fit$Vp[1:Nfixed, 1:Nfixed]
```

We now extract the estimated quantiles evaluated at \(\texttt{cca20}\) and \(\texttt{cca80}\) (without random effects), namely \[
\widehat Q^\tau\_{20}(t) = \alpha^\tau(t) + \int\_\mathcal{S} \text{cca}\_{20}(s)\hat\beta^\tau(s,t) ds
,\quad
\widehat Q^\tau\_{80}(t) = \alpha^\tau(t) + \int\_\mathcal{S} \text{cca}\_{80}(s)\hat\beta^\tau(s,t) ds,
\]

and their difference \(\widehat D^\tau(t) = \widehat Q^\tau\_{20}(t) -\widehat Q^\tau\_{80}(t)\). Notice the multiplication by S in the definition of Xobs in nd20 and nd80. The model-based standard errors associated to the differences are also computed (and used for model-based confidence intervals later).

```
pred20 <- pred80 <- predDiff <- sd.par <- rep(NA, maxT)

for(t in 1:maxT){
  
  ## New datasets for prediction (one for each longitudinal time stamp)
  nd20 <- data.frame(time = rep(t,S),tMat=rep(t,S), sMat=sVec, Xobs=cca20*S)
  nd80 <- data.frame(time = rep(t,S),tMat=rep(t,S), sMat=sVec, Xobs=cca80*S)
  
  ## Predictions 
  pred20[t] <- mean(predict(fit,  exclude='s(id)', newdata=nd20,newdata.guaranteed=TRUE))
  pred80[t] <- mean(predict(fit,  exclude='s(id)', newdata=nd80,newdata.guaranteed=TRUE))
  predDiff[t] <- pred20[t] - pred80[t]
  
  ## Computation of parametric standard errors
  ## Notice that we select the part of the covariance matrix that does not include the random intercepts, 
  ## so only the columns related to fixed effects (via Nfixed)
  pred20.mat <- predict(fit,  exclude='s(id)', type='lpmatrix',newdata=nd20,newdata.guaranteed=TRUE)[,1:Nfixed]
  pred80.mat <- predict(fit,  exclude='s(id)', type='lpmatrix',newdata=nd80,newdata.guaranteed=TRUE)[,1:Nfixed]
  
  A <- colMeans(pred20.mat) - colMeans(pred80.mat)
  A <- as.matrix(A)
  sd.par[t] <- sqrt(t(A)%*%cov%*%A)
}
```

The plots below show the estimated quantile profiles. They turn out to be linear in longitudinal time. This is not due to constraints in the model, but to penalization of non-linearity.

```
data.plot <- data.frame(pred=c(pred20,pred80),
                        t=rep(1:maxT, 2),
                        nb=factor(rep(1:2,each=maxT)))

plot.pred.2080 <- ggplot(data.plot, aes(x=t,y=pred,group=nb, color=nb)) + geom_line()  + theme(legend.position="bottom") + labs(color = "") + ylab("") + scale_color_manual(values=c( "1"="blue", "2"="red"), labels=c(expression(paste(widehat(Q)[20]^tau,"(t)")),expression(paste(widehat(Q)[80]^tau,"(t)"))))

data.plot <- data.frame(pred=predDiff,
                        t=1:maxT)

plot.pred.diff <- ggplot(data.plot, aes(x=t,y=pred))+ geom_line()+ ylab(expression(paste(widehat(D)^tau,"(t)")))

grid.arrange(plot.pred.2080, plot.pred.diff, ncol=2)
```

## Bootstrap

We now run the two bootstrap schemes considered in our paper, namely block and wild bootstrap. If you run the code yourself, then we suggest to first decrease the number of bootstrap samples.

```
source("bootstrap_functions.R")

# Number of bootstrap samples
B <- 100
```

### Block bootstrap

```
blockb_seed <- 1234

## Block bootstrap
bb <- boot_pred_st_block(d=myDataFit, B=B, seed=blockb_seed, tau=tau, X20=cca20, X80=cca80, model = fit)
```

The object bb contains the estimated quantiles and their differences, estimated from the bootstrap samples as well as some info about the bootstrap procedure. For example, pred20 is the \(B\times \texttt{tMax}\) matrix of bootstrap estimates of quantiles for cca20 (tMax=1570, the maximum time of observation).

```
names(bb)
```

```
## [1] "pred20"       "pred80"       "predDiff"     "seed"         "d"           
## [6] "tau"          "running_time"
```

```
dim(bb$pred20)
```

```
## [1]  100 1570
```

The following plots show the block bootstrap estimated quantiles (in grey) against \(\widehat Q^\tau\_{20}\) and \(\widehat Q^\tau\_{80}\), in blue and red respectively. Moreover, we show \(\widehat D^\tau(t)\) (in black) with the block bootstrap estimates (in grey).

```
pred20.bb <- pred80.bb <- predDiff.bb <- nb <- t <- NULL
for(i in 1:B){
  pred20.bb <- c(pred20.bb, bb$pred20[i,])
  pred80.bb <- c(pred80.bb, bb$pred80[i,])
  predDiff.bb <- c(predDiff.bb, bb$predDiff[i,])
  nb <- c(nb, rep(i, maxT))
  t <- c(t, 1:maxT)
}

data.plot.p20 <- data.frame(p20=c(pred20.bb,pred20),
                        nb=factor(c(nb, rep((i+1),maxT))),
                        t=c(t,1:maxT),
                        curve=factor(c(rep(1,(B*maxT)), rep(2,maxT)))
)


data.plot.p80 <- data.frame(p80=c(pred80.bb,pred80),
                        nb=factor(c(nb, rep((i+1),maxT))),
                        t=c(t,1:maxT),
                        curve=factor(c(rep(1,(B*maxT)), rep(2,maxT)))
)

ymin <- min(min(data.plot.p20$p20), min(data.plot.p80$p80))
ymax <- max(max(data.plot.p20$p20), max(data.plot.p80$p80))

plot.20.bb <- ggplot(data.plot.p20, aes(x=t,y=p20,group=nb, color=curve)) + geom_line()   + ylab(expression(paste(widehat(Q)[20]^tau,"(t)"))) +  theme(legend.position="") + scale_color_manual(values=c( "1"="grey", "2"="blue")) + ylim(ymin, ymax)
 
plot.80.bb <- ggplot(data.plot.p80, aes(x=t,y=p80,group=nb, color=curve)) + geom_line()   + ylab(expression(paste(widehat(Q)[80]^tau,"(t)"))) +  theme(legend.position="") + scale_color_manual(values=c( "1"="grey", "2"="red")) + ylim(ymin, ymax)


data.plot.pd <- data.frame(pd=c(predDiff.bb,predDiff),
                        nb=factor(c(nb, rep(i+1,maxT))),
                        t=c(t,1:maxT),
                        curve=factor(c(rep(1,(B*maxT)), rep(2,maxT)))
)


plot.diff.bb <- ggplot(data.plot.pd, aes(x=t,y=pd, group=nb, color=curve)) + geom_line()   + ylab(expression(paste(widehat(D)^tau,"(t)"))) +  theme(legend.position="") + scale_color_manual(values=c( "1"="grey", "2"="black")) 


grid.arrange(plot.20.bb, plot.80.bb, plot.diff.bb, nrow=1)
```

### Wild bootstrap

```
wildb_seed <- 4321

## Wild bootstrap
wb <- boot_pred_st_wild(d=myDataFit, B=B, seed=wildb_seed, tau=tau, X20=cca20, X80=cca80, model = fit)
```

The structure of wb is exactly the same as for bb. We make the same plots as in the case of block bootstrap.

```
pred20.wb <- pred80.wb <- predDiff.wb <- nb <- t <- NULL
for(i in 1:B){
  pred20.wb <- c(pred20.wb, wb$pred20[i,])
  pred80.wb <- c(pred80.wb, wb$pred80[i,])
  predDiff.wb <- c(predDiff.wb, wb$predDiff[i,])
  nb <- c(nb, rep(i, maxT))
  t <- c(t, 1:maxT)
}

data.plot.p20 <- data.frame(p20=c(pred20.wb,pred20),
                        nb=factor(c(nb, rep(i+1,maxT))),
                        t=c(t,1:maxT),
                        curve=factor(c(rep(1,(B*maxT)), rep(2,maxT)))
)


data.plot.p80 <- data.frame(p80=c(pred80.wb,pred80),
                        nb=factor(c(nb, rep(i+1,maxT))),
                        t=c(t,1:maxT),
                        curve=factor(c(rep(1,(B*maxT)), rep(2,maxT)))
)

ymin <- min(min(data.plot.p20$p20), min(data.plot.p80$p80))
ymax <- max(max(data.plot.p20$p20), max(data.plot.p80$p80))

plot.20.wb <- ggplot(data.plot.p20, aes(x=t,y=p20,group=nb, color=curve)) + geom_line()   + ylab(expression(paste(widehat(Q)[20]^tau,"(t)"))) +  theme(legend.position="") + scale_color_manual(values=c( "1"="grey", "2"="blue")) + ylim(ymin, ymax)
 
plot.80.wb <- ggplot(data.plot.p80, aes(x=t,y=p80,group=nb, color=curve)) + geom_line()   + ylab(expression(paste(widehat(Q)[80]^tau,"(t)"))) +  theme(legend.position="") + scale_color_manual(values=c( "1"="grey", "2"="red")) + ylim(ymin, ymax)


data.plot.pd <- data.frame(pd=c(predDiff.wb,predDiff),
                        nb=factor(c(nb, rep(i+1,maxT))),
                        t=c(t,1:maxT),
                        curve=factor(c(rep(1,(B*maxT)), rep(2,maxT)))
)


plot.diff.wb <- ggplot(data.plot.pd, aes(x=t,y=pd, group=nb, color=curve)) + geom_line()   + ylab(expression(paste(widehat(D)^tau,"(t)"))) +  theme(legend.position="") + scale_color_manual(values=c( "1"="grey", "2"="black")) 


grid.arrange(plot.20.wb, plot.80.wb, plot.diff.wb, nrow=1)
```

## Bias-adjustment and confidence intervals

We finally plot the parametric pointwise confidence bands for \(\widehat \Delta^\tau(t)\) (in black), as well as its bias-adjusted version with bootstrap-based confidence bands (in orange), as we do in our data application in the manuscript.

```
diff.adj <- matrix(NA, B,maxT)

for(i in 1:B){
  diff.adj[i,] <- wb$predDiff[i,] - predDiff
 }

## Computation of bias
bias.wb <- apply(diff.adj,2,mean)

## Bias-adjusted prediction by means of wild bootstrap
pred.adj.wb <- predDiff - bias.wb

## Estimated standard error from block bootstrap
sd.bb <- apply(bb$predDiff,2,sd)
```

```
data.plot <- data.frame(diff=predDiff, 
                        low2 =predDiff - 1.96*sd.par ,up2 =predDiff + 1.96*sd.par ,
                       diff_boot =  pred.adj.wb, 
                       low_boot = pred.adj.wb - 1.96*sd.bb ,up_boot= pred.adj.wb + 1.96*sd.bb ,
                        time=1:maxT)


ggplot(data.plot, aes(x=time, y=diff))  + geom_line(lwd=1) + geom_line(aes(x=time, y=diff_boot), color="orange",lwd=1)  + geom_ribbon(aes(ymin=low2, ymax=up2, col='black'), alpha=0.2, linetype=2,lwd=1, show.legend = TRUE) +
  geom_ribbon(aes(ymin=low_boot, ymax=up_boot, col='orange'), alpha=0.2, linetype=2,lwd=1, show.legend = TRUE) +xlab("t")  + labs(title = "")+ scale_color_manual( "",labels =  c( "Model-based","Bootstrap") , values=c('black' ='black', "orange" = "orange"))+ theme(legend.position="bottom")+ ylab(expression(paste(hat(D)^tau,"(t)")))
```

## Fitting a time-homogeneous model

In the manuscript we also wotk with a time-homogeneous model, namely

\[Q\_{Y\_{ij}| {X}\_{ij}, u\_i}^\tau = \alpha^\tau + \int\_{\mathcal{S}} \beta^\tau(s) {X}\_{ij}(s)ds + u\_i,\] The syntax for the model is a simplified version of the one used for the time-inhomogeneous model. The model does not appear to be relevant for the DTI data, so we do examine the model fit.

```
## Time-homogeneous model
formula.th <- as.formula('y ~ s(sMat, by=Xobs, bs="cr", k=10) + s(id, bs="re")')
fit.th <- qgam(formula.th, qu=tau, data=myDataFit)
```

Finally, we point out that it is possible to let the learning rate of vary with the covariates with the following syntax.

```
formula.th1 <- as.formula('~ s(sMat, by=Xobs, bs="cr", k=10) + s(id, bs="re")')
fit.th.flex <- qgam(list(formula.th, formula.th1), qu=tau, data=myDataFit)
```

This allows for more flexibility in the estimation, and it was used in the computations presented in our paper.
