# Supplementary material for: Quantile Regression for Longitudinal Functional Data with Application to Feed Intake of Lactating Sows
Source: J Agric Biol Environ Stat. 2024 Feb 6;30(1):211–30. doi: 10.1007/s13253-024-00601-5 (PMC11885350; doi:10.1007/s13253-024-00601-5)
Supplement: Supplementary file 2 — (zip 1175 KB) [file 13253_2024_601_MOESM2_ESM.zip › Revised supplementary/qgam_vs_aqmm_all_scenarios_conditional_and_marginal.pdf]

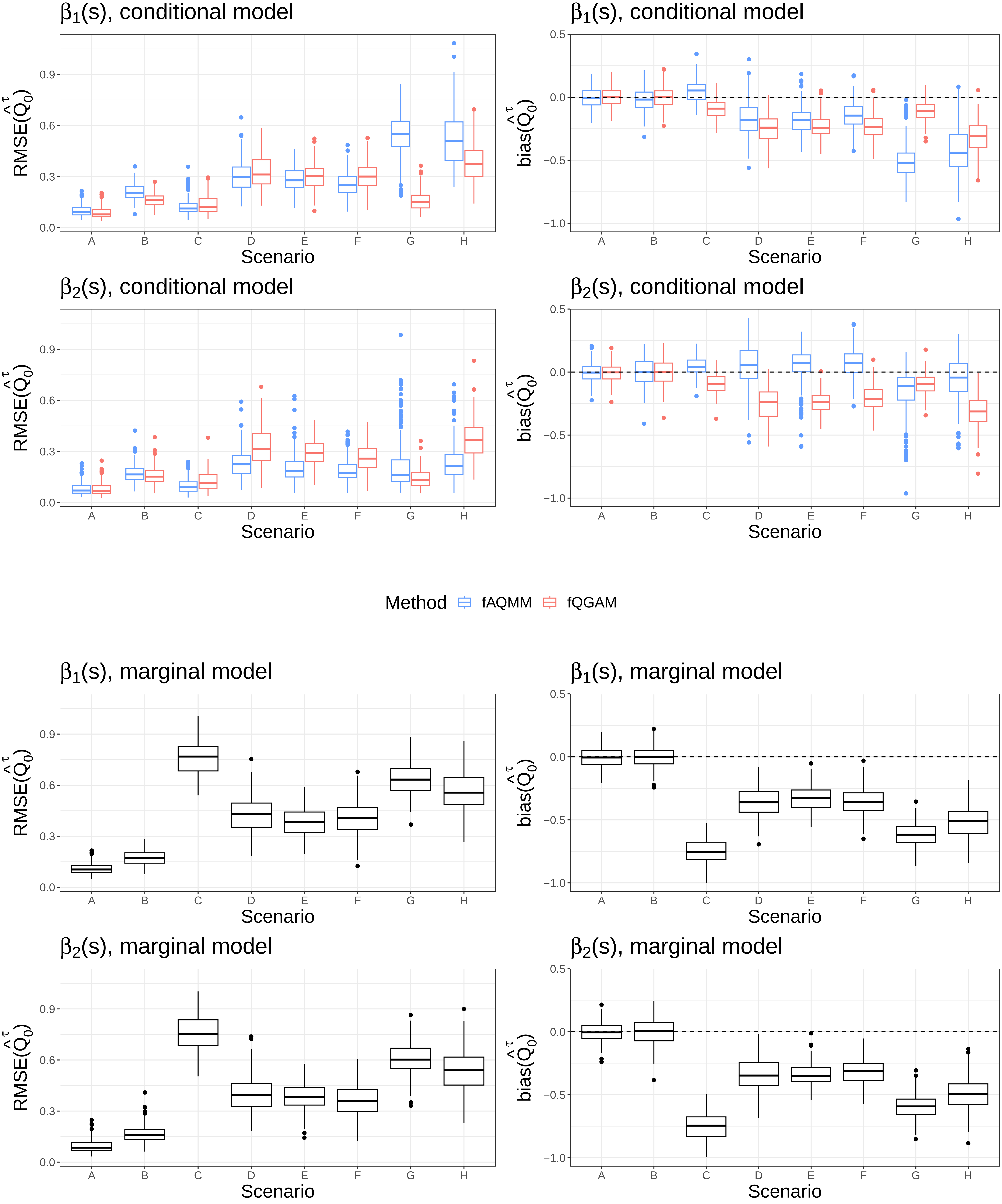

β1(s), conditional model
β1(s), conditional model
β2(s), conditional model
β2(s), conditional model
β1(s), marginal model
β1(s), marginal model
β2(s), marginal model
β2(s), marginal model
RMSE(Q̂0τ)
bias(Q̂0τ)
0.0
0.3
0.6
0.9
0.5
0.0
−0.5
−1.0
A
B
C
D
E
F
G
H
Scenario
Method
fAQMM
fQGAM
