# Supplementary figures and images for: Quantile Regression for Longitudinal Functional Data with Application to Feed Intake of Lactating Sows
Source: J Agric Biol Environ Stat. 2024 Feb 6;30(1):211–30. doi: 10.1007/s13253-024-00601-5 (PMC11885350; doi:10.1007/s13253-024-00601-5)

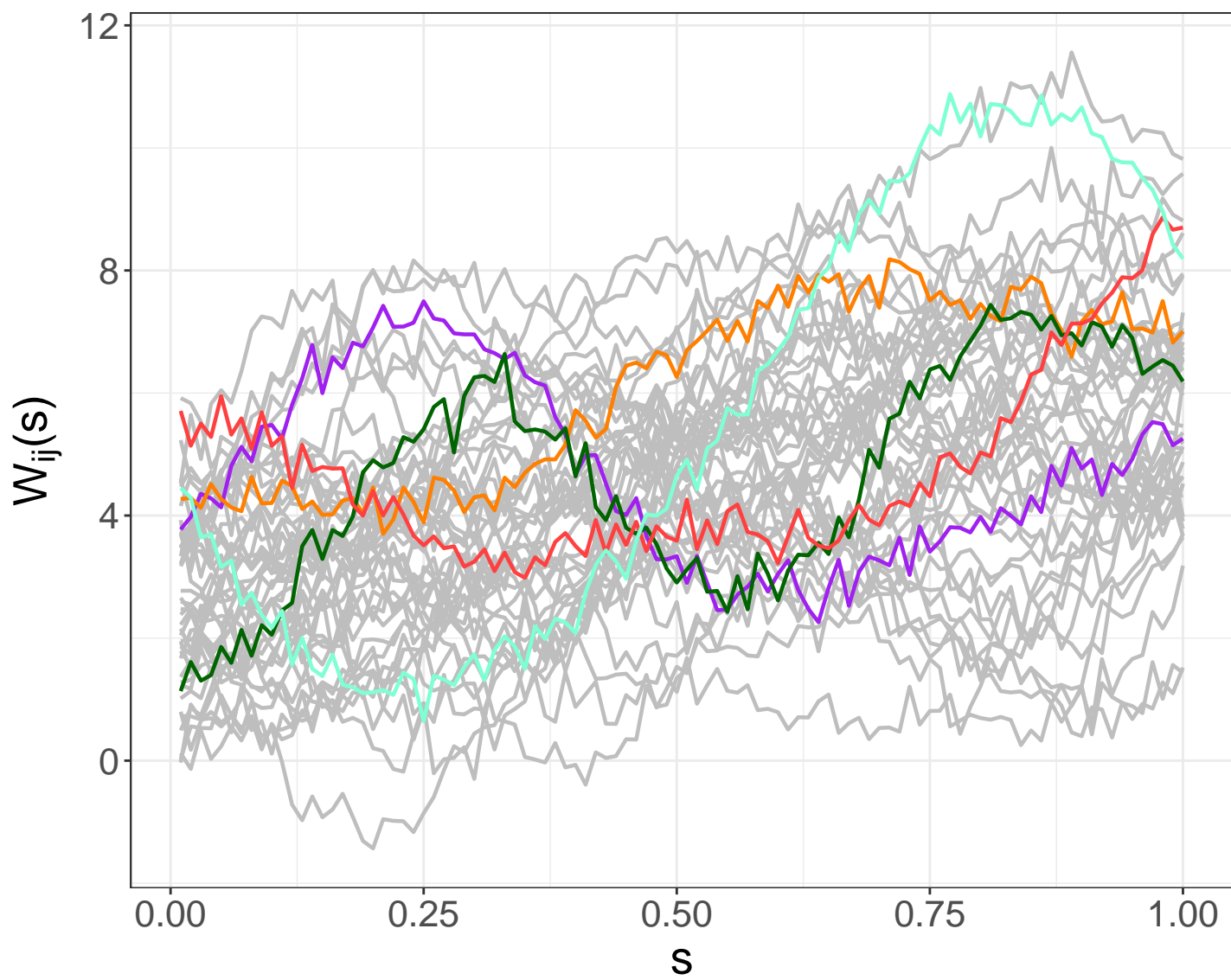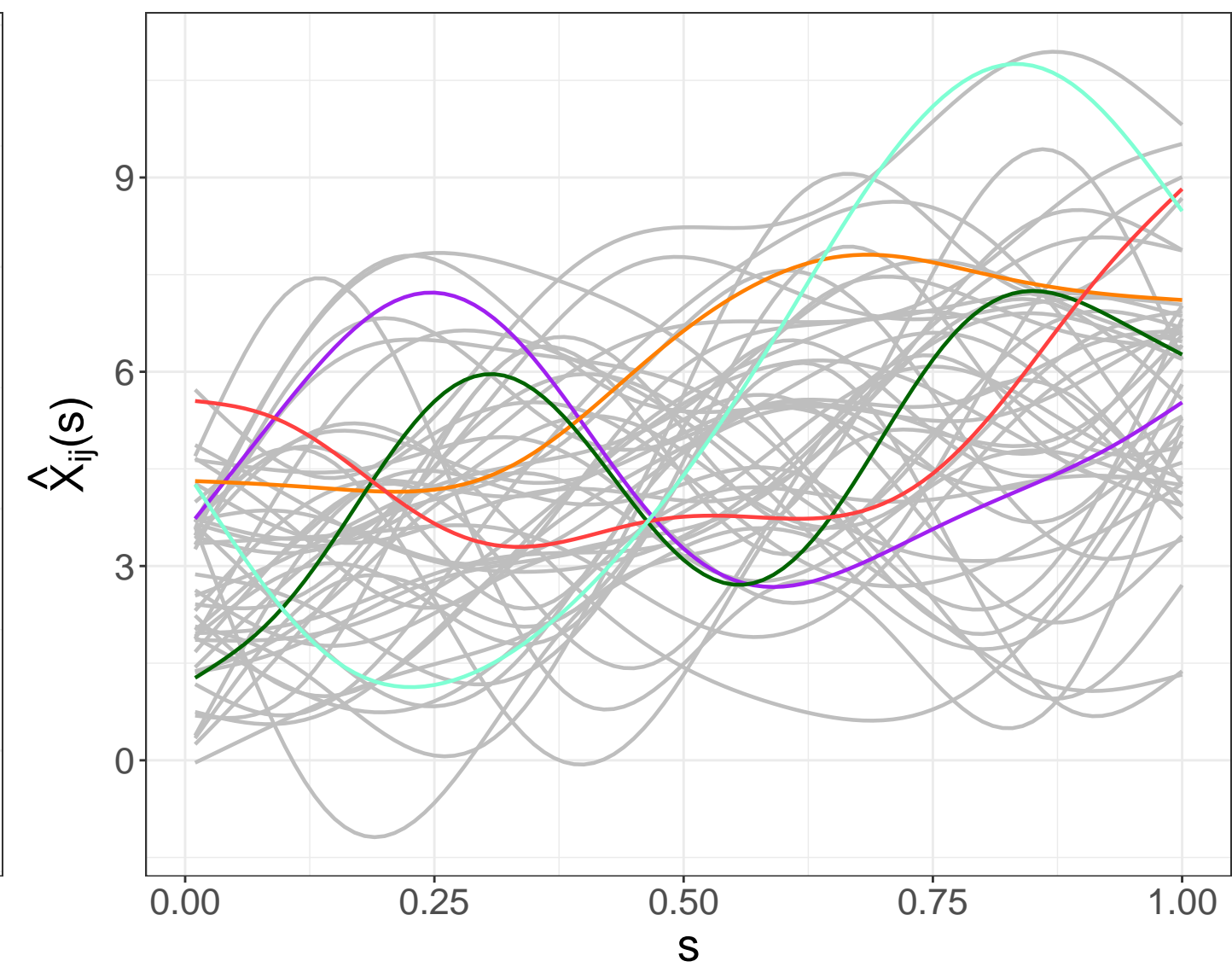

Supplement: Supplementary file 2 — (zip 1175 KB) [file 13253_2024_601_MOESM2_ESM.zip › Revised supplementary/curves_example.pdf]

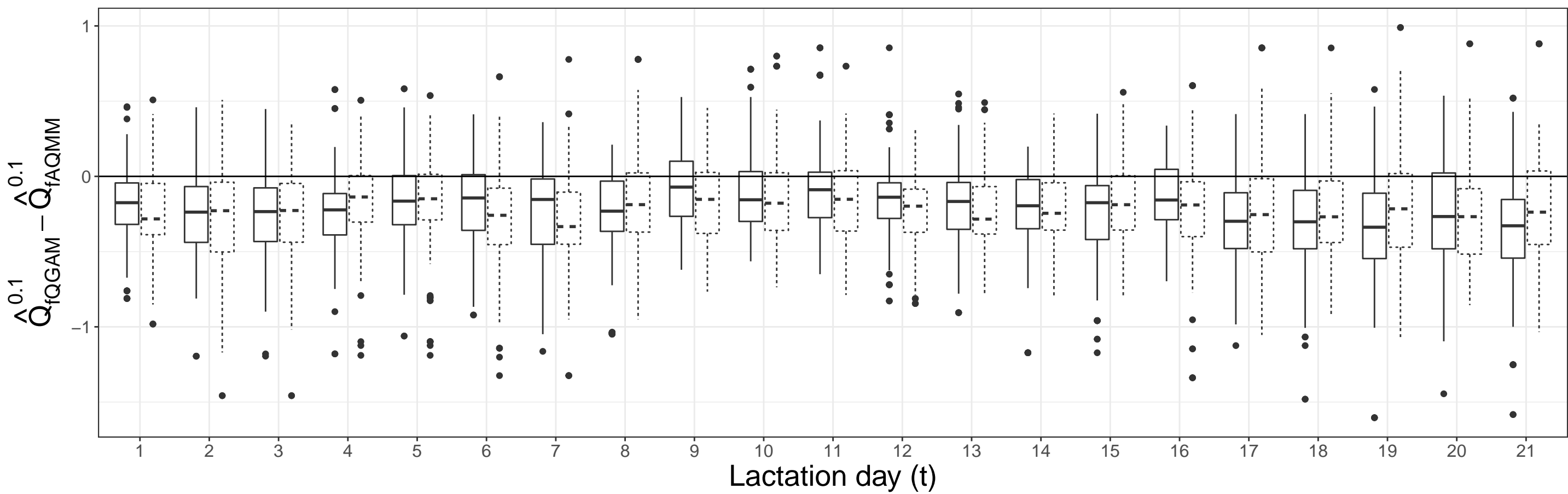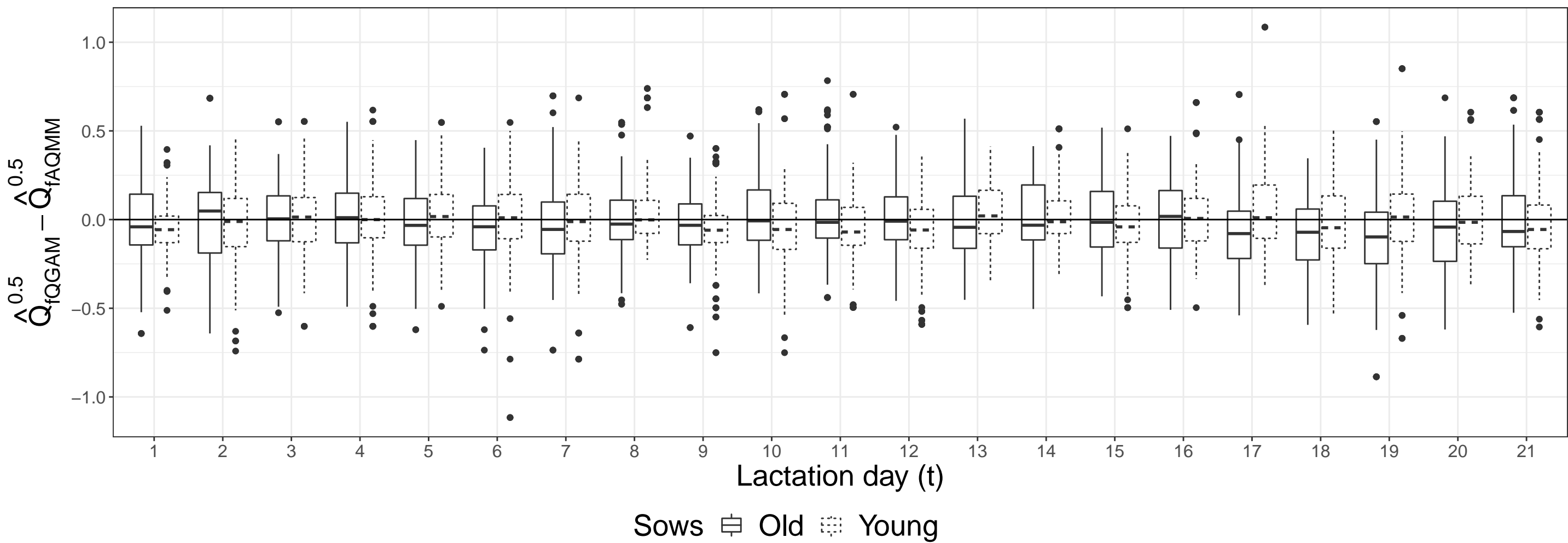

Supplement: Supplementary file 2 — (zip 1175 KB) [file 13253_2024_601_MOESM2_ESM.zip › Revised supplementary/boxplot_difference_linpred_methods.pdf]

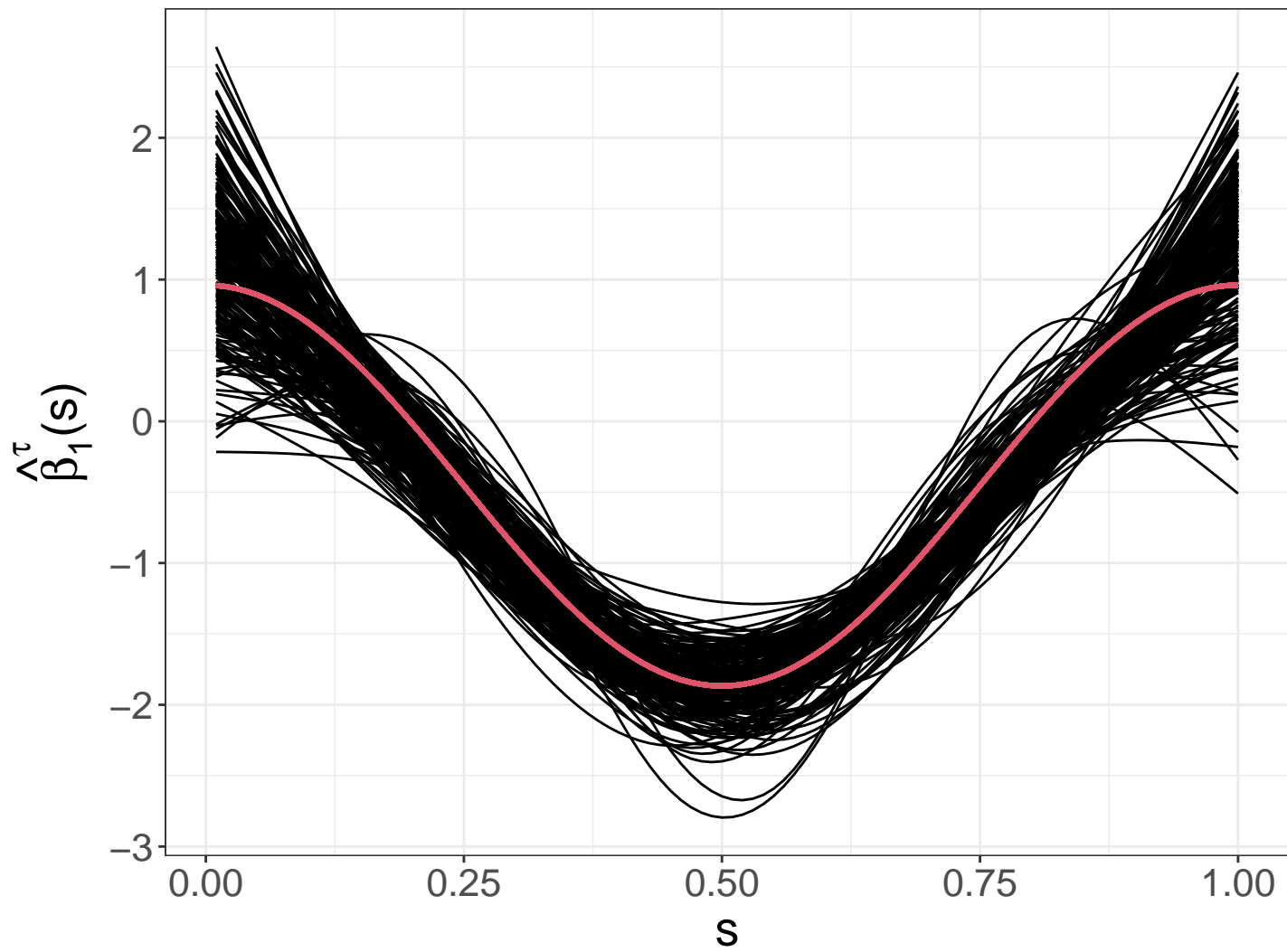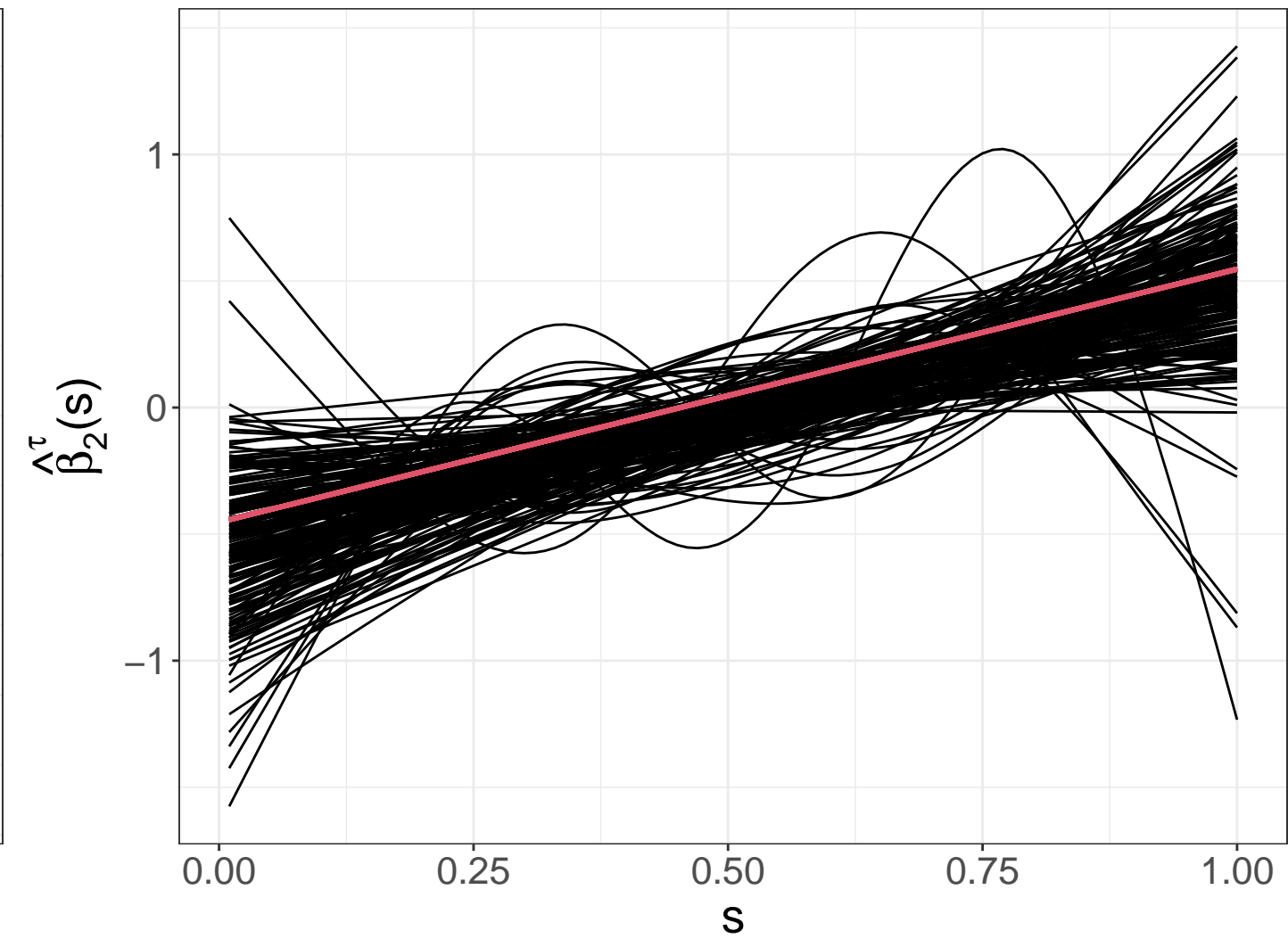

Supplement: Supplementary file 2 — (zip 1175 KB) [file 13253_2024_601_MOESM2_ESM.zip › Revised supplementary/comp_beta1s_beta2s.pdf]

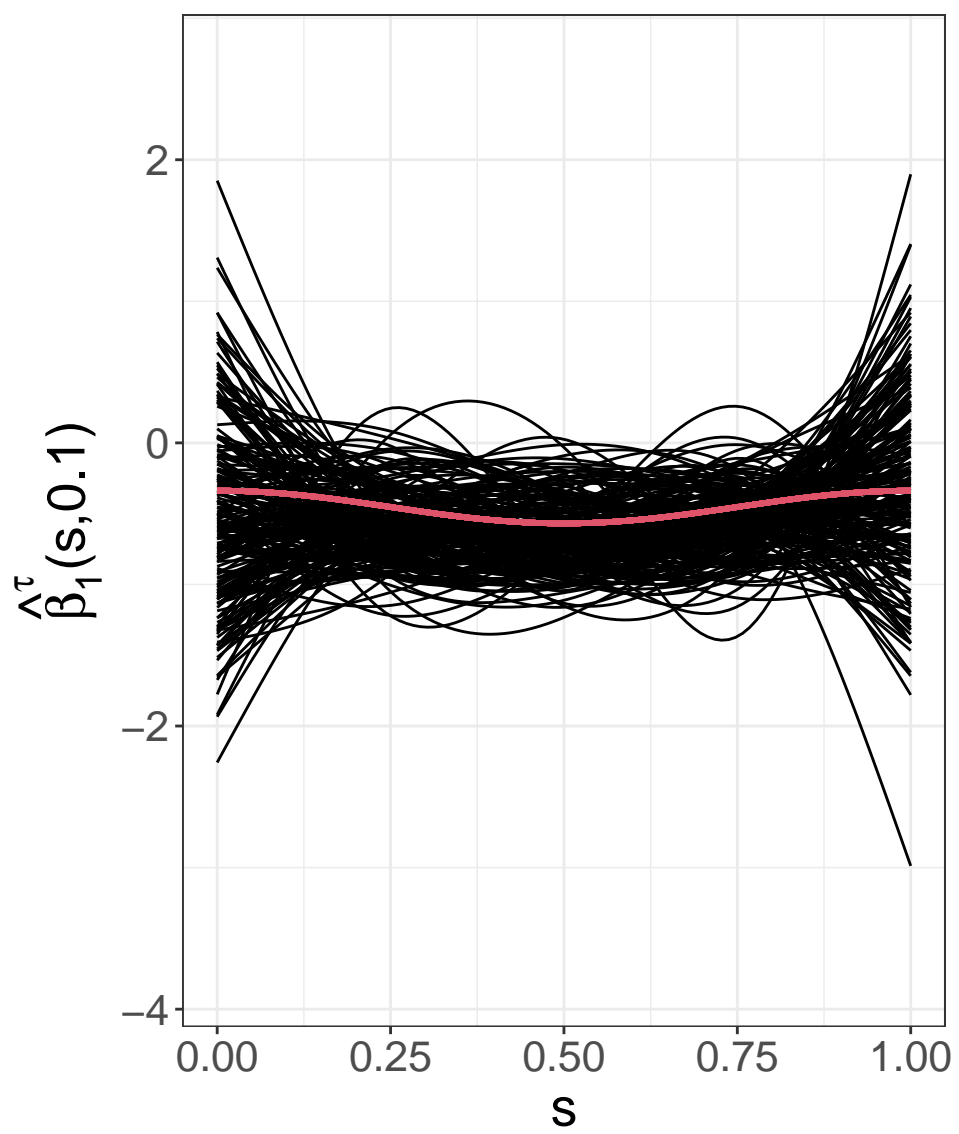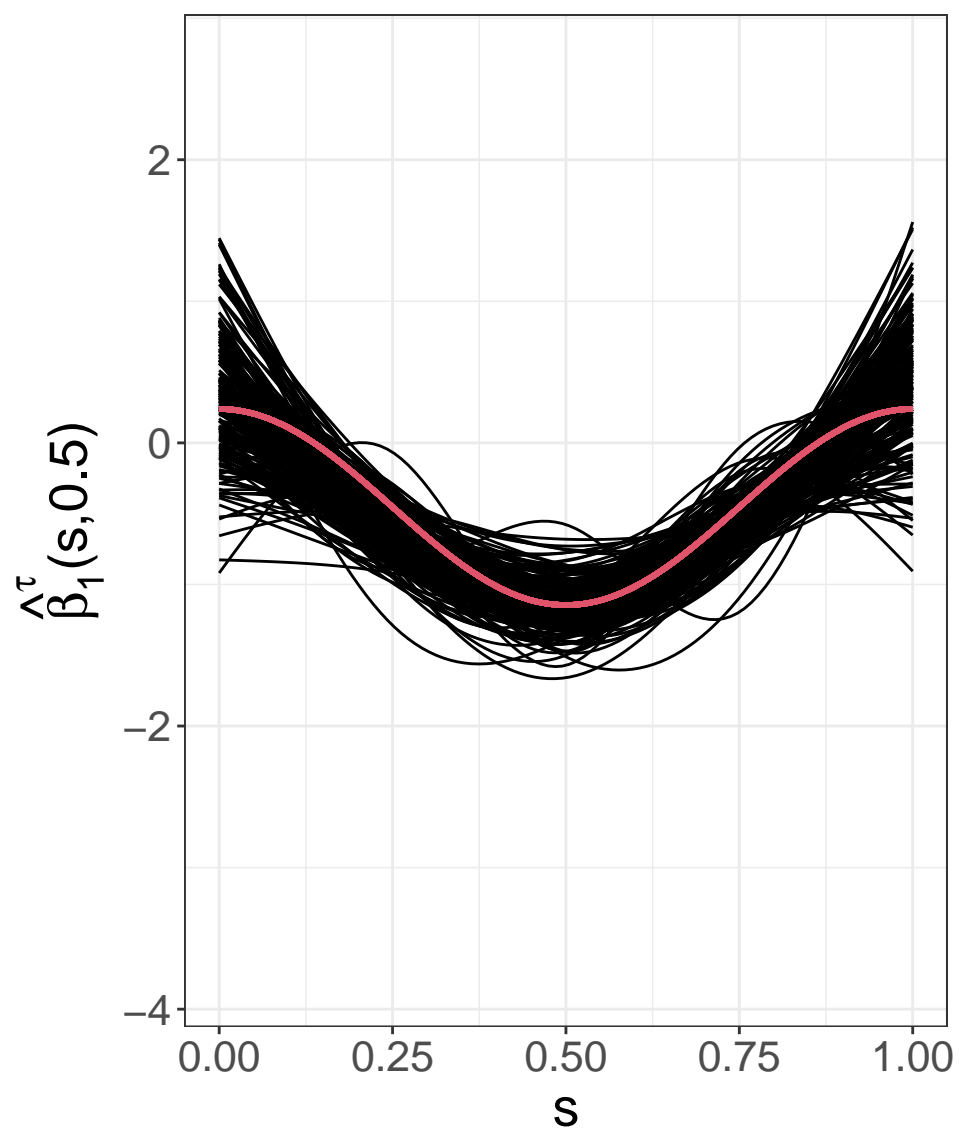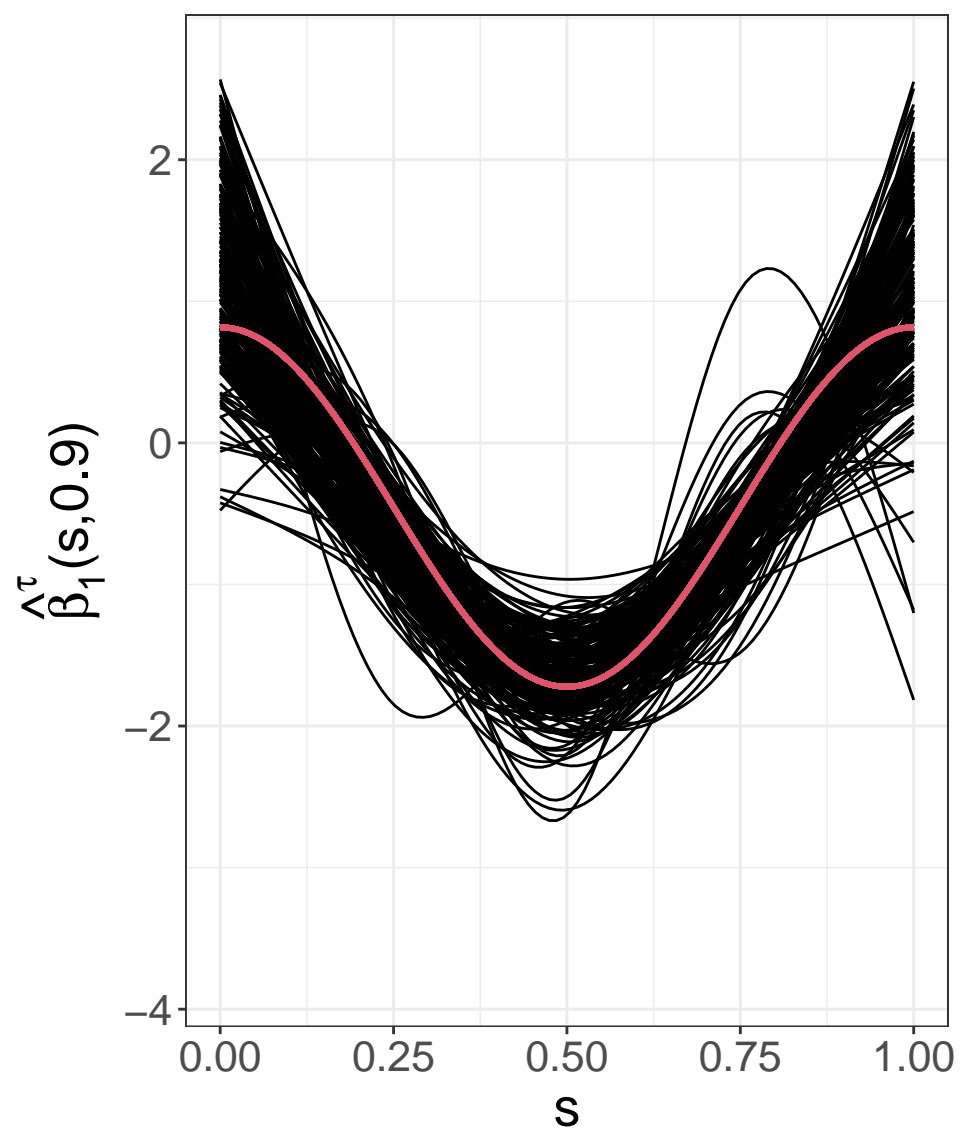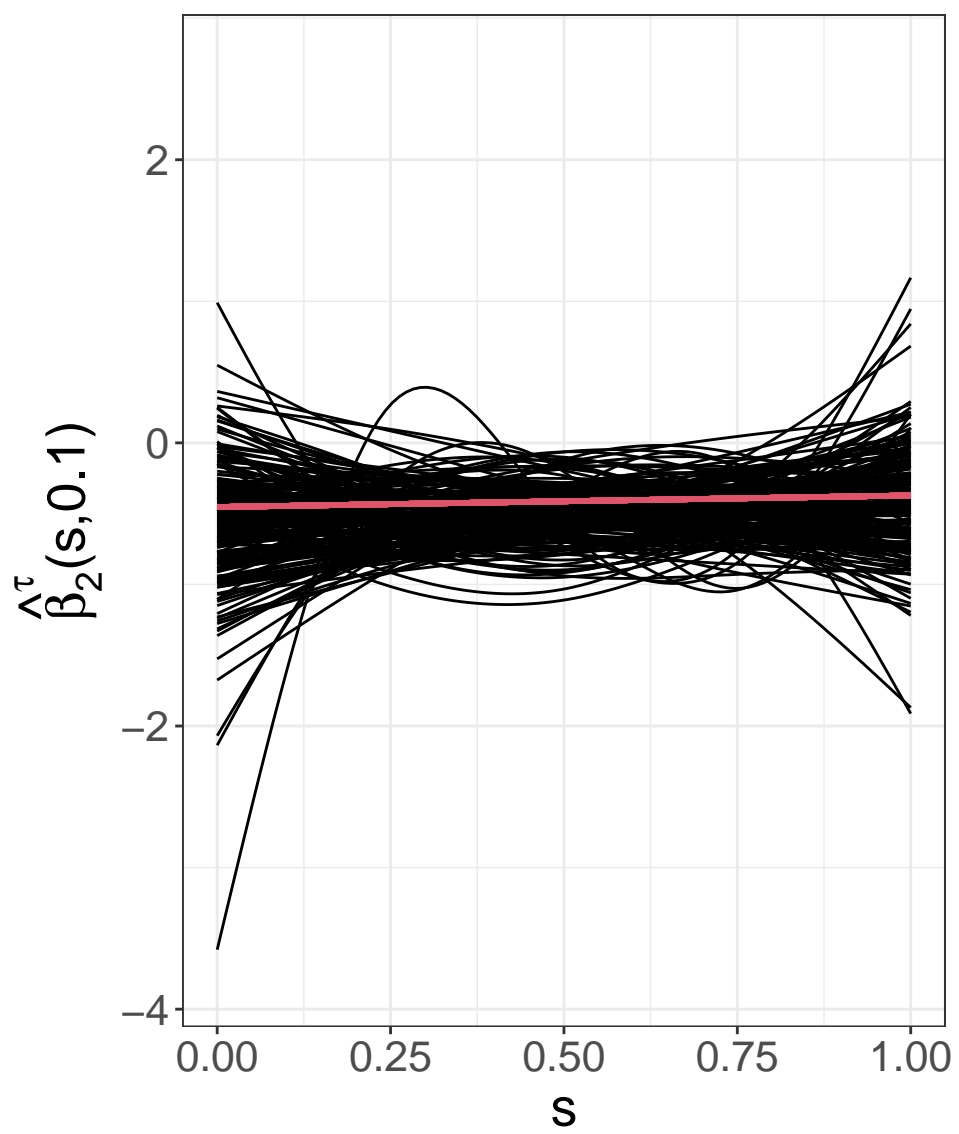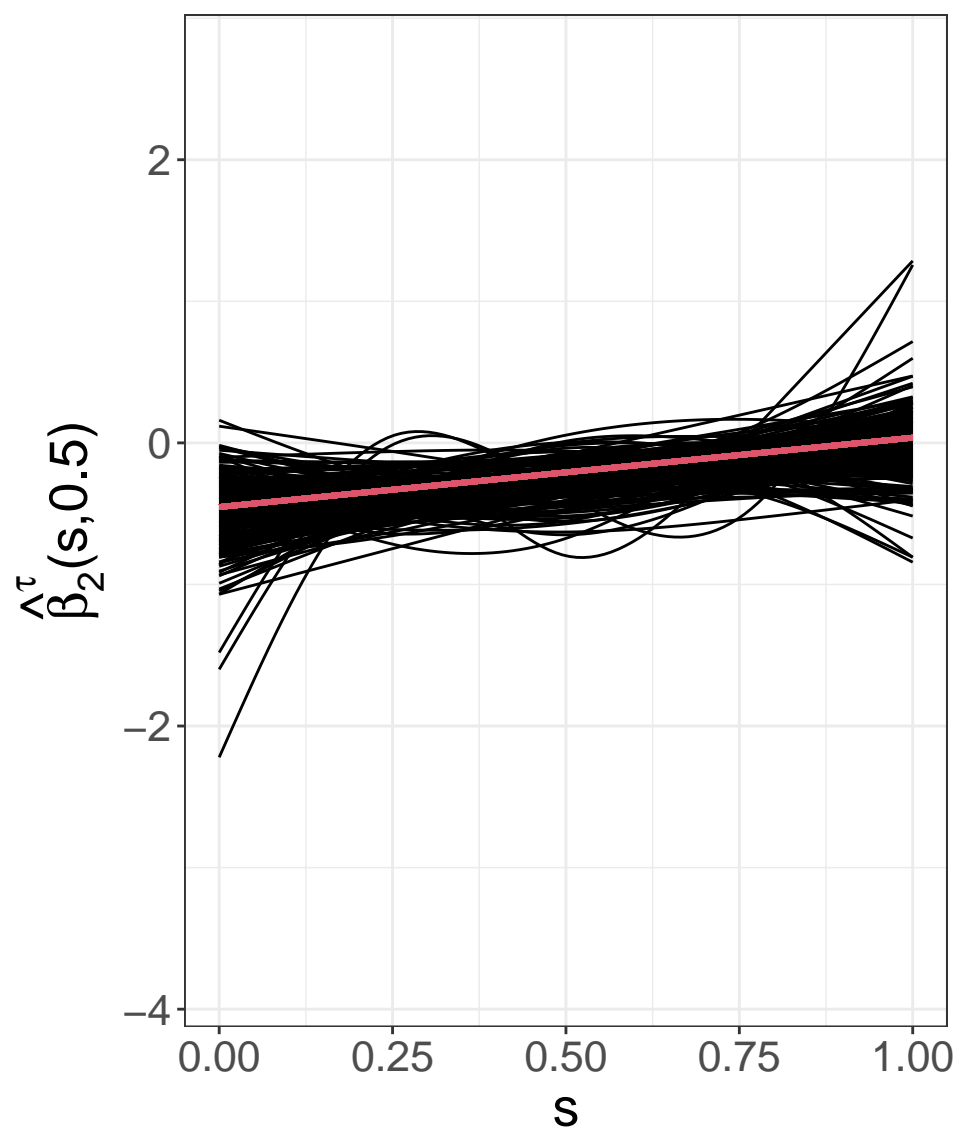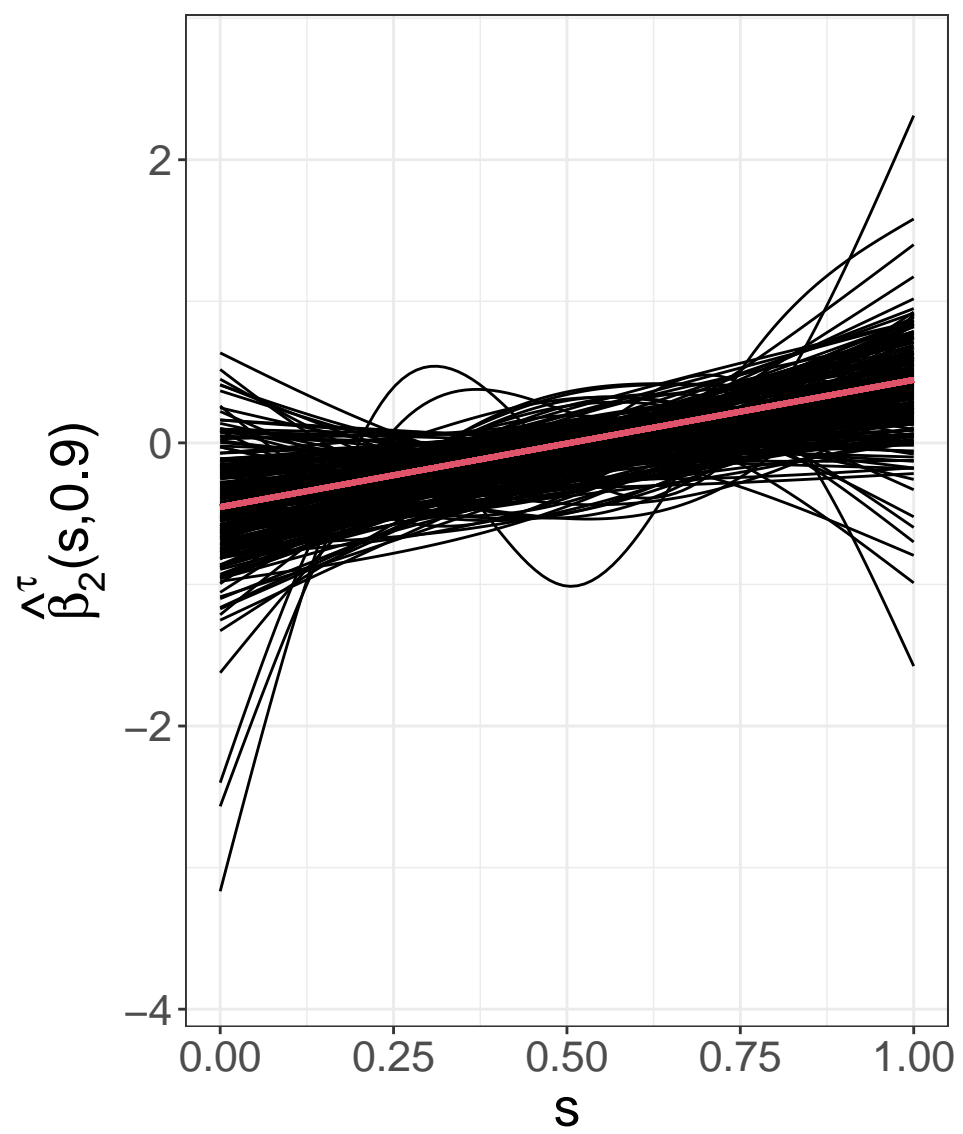

Supplement: Supplementary file 2 — (zip 1175 KB) [file 13253_2024_601_MOESM2_ESM.zip › Revised supplementary/betast12_different_times.pdf]

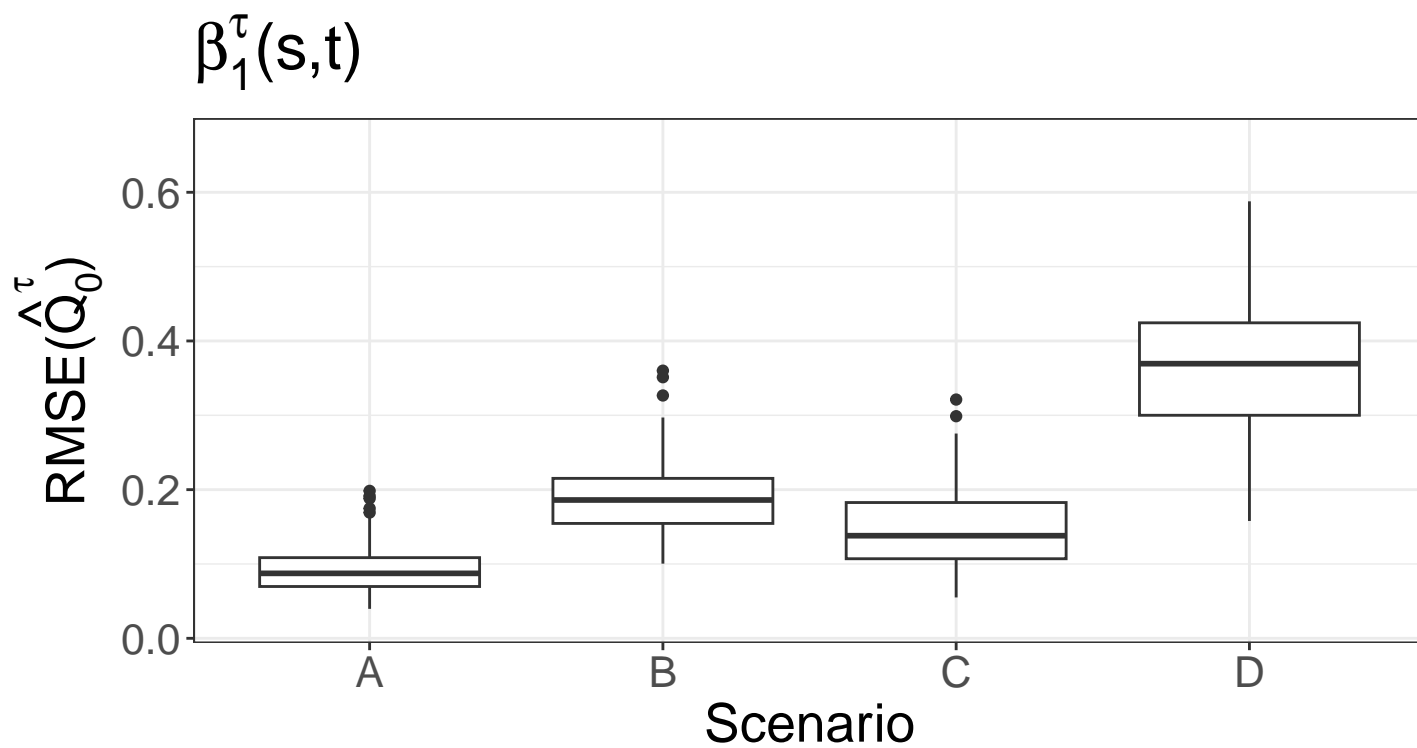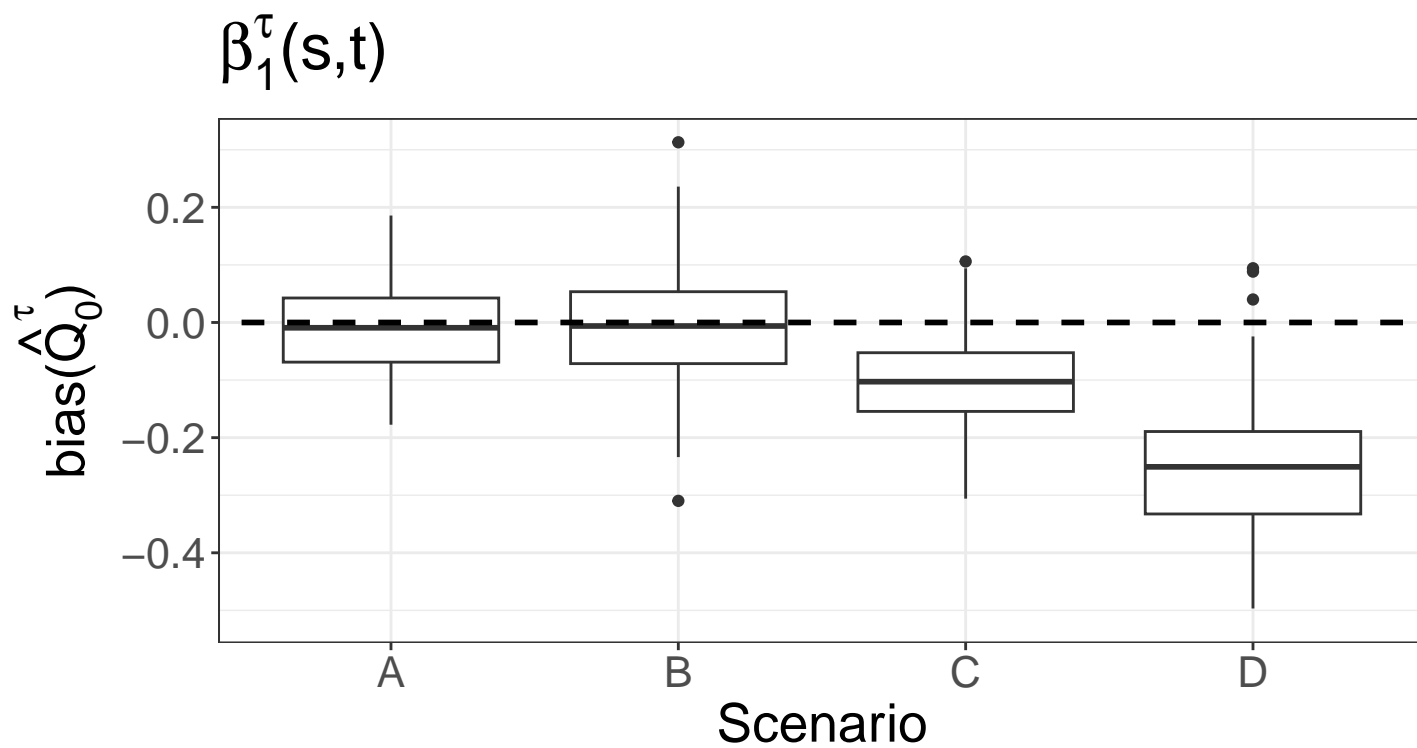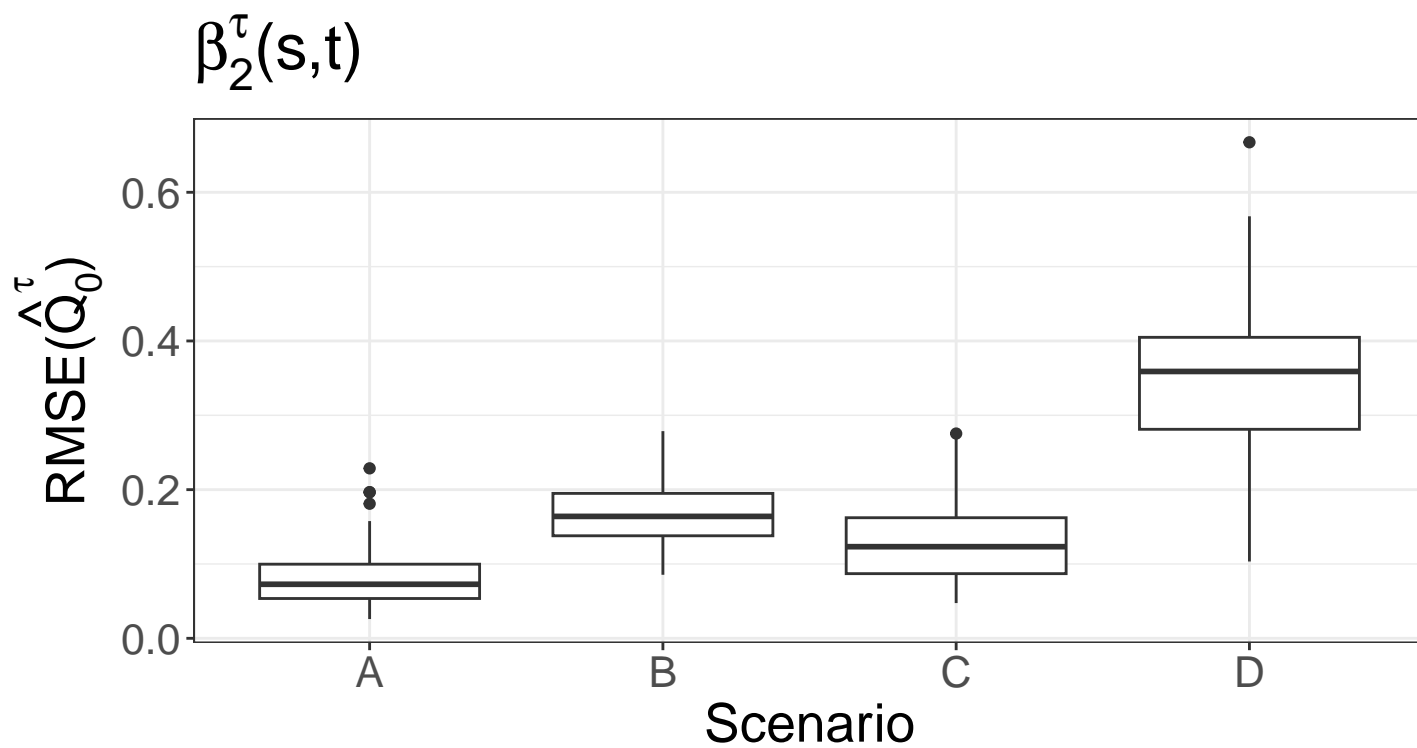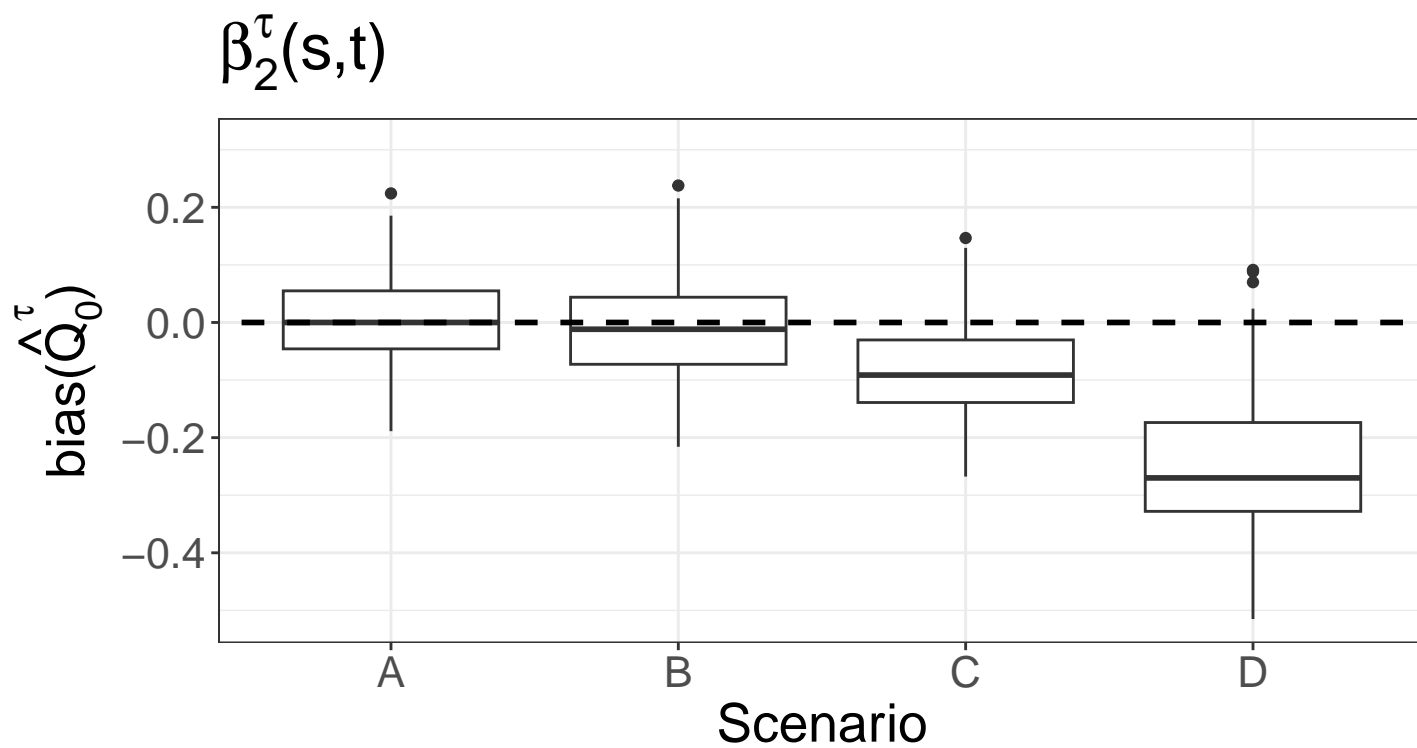

Supplement: Supplementary file 2 — (zip 1175 KB) [file 13253_2024_601_MOESM2_ESM.zip › Revised supplementary/comp_betast1_vs_betast2_Qerrors.pdf]

Block resampling bootstrap

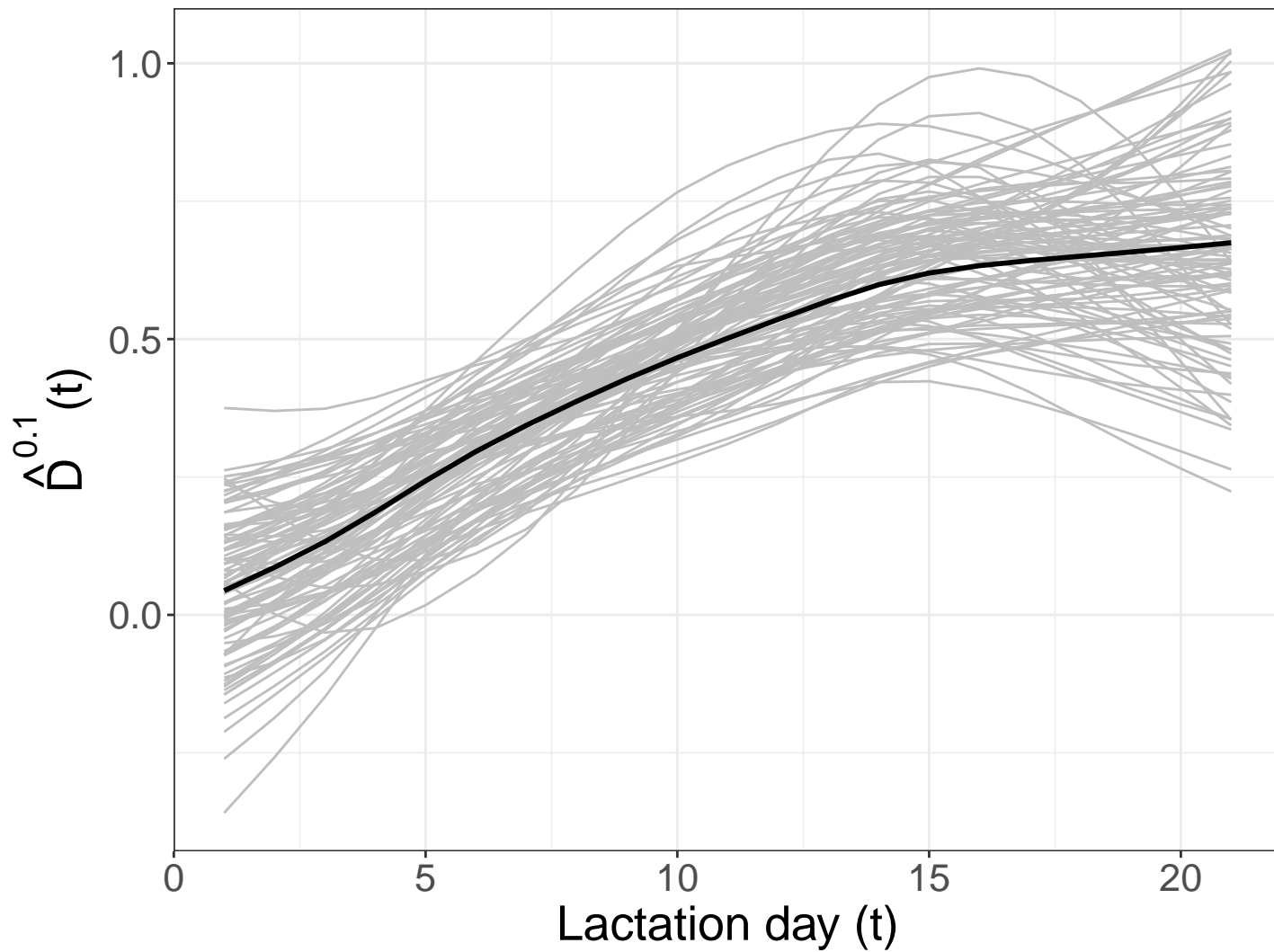

Wild bootstrap

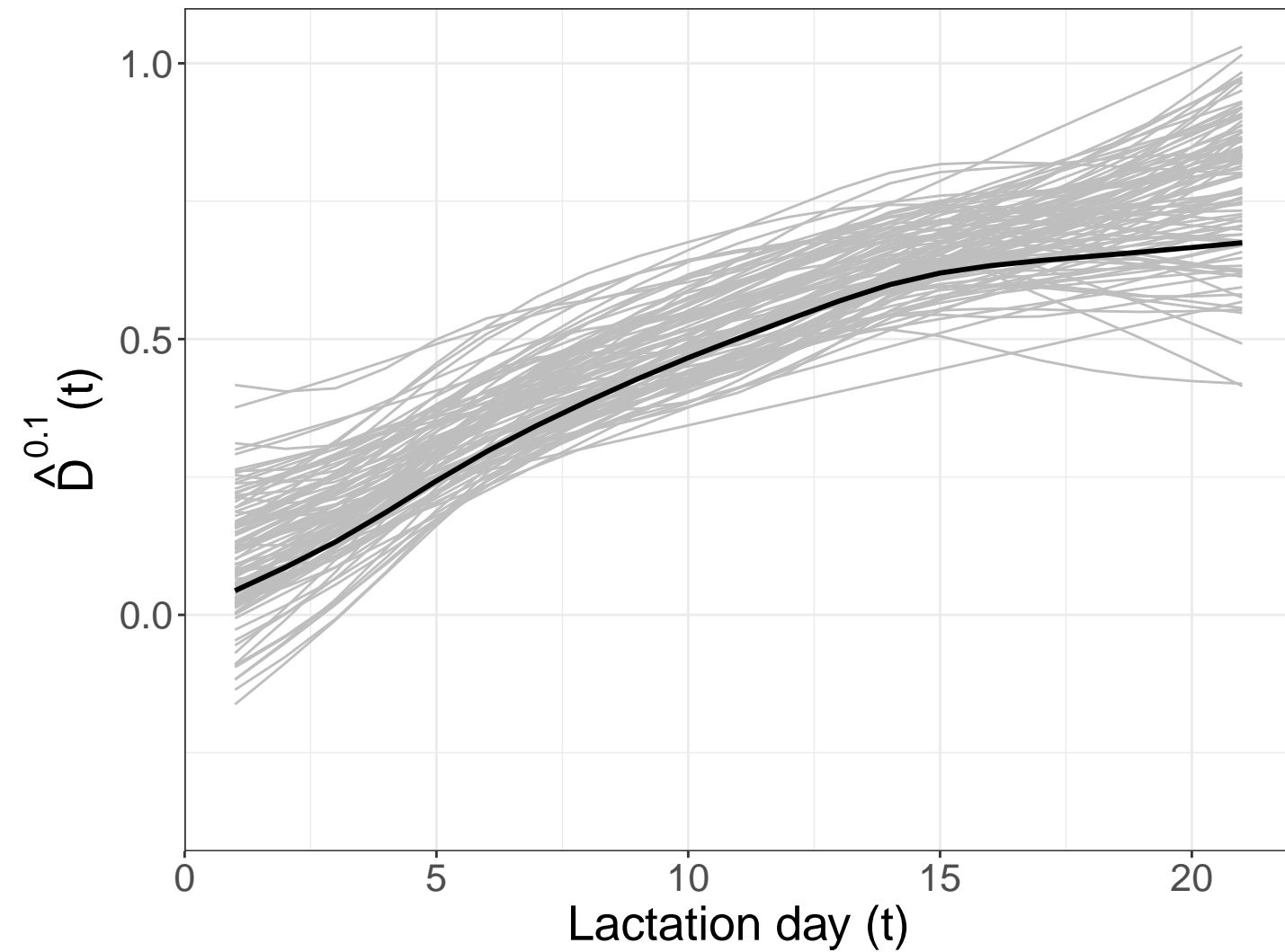

Supplement: Supplementary file 2 — (zip 1175 KB) [file 13253_2024_601_MOESM2_ESM.zip › Revised supplementary/pred_Diff_model_vs_boot.pdf]
